# Supplementary material for: How spin relaxes and dephases in bulk halide perovskites
Source: Nat Commun. 2024 Jan 2;15:188. doi: 10.1038/s41467-023-42835-w (PMC10761878; doi:10.1038/s41467-023-42835-w)
Supplement: Supplementary file 1 — Supplementary Information [file 41467_2023_42835_MOESM1_ESM.pdf]

Supplementary Information for: How Spin Relaxes and Dephases in Bulk Halide Perovskites

Junqing Xu[a],[1,2] Kejun Li[a],[3] Uyen Huynh,[4] Mayada Fadel,[5] Jinsong Huang,[6] Ravishankar Sundararaman,[5,*] Valy Vardeny,[4] and Yuan Ping[7,3,†]

[1] *Department of Physics, Hefei University of Technology, Hefei, Anhui, China*
[2] *Department of Chemistry and Biochemistry, University of California, Santa Cruz, California, 95064, USA*
[3] *Department of Physics, University of California, Santa Cruz, California, 95064, USA*
[4] *Physics Department, University of Utah, 115 South 1400 East*
[5] *Department of Materials Science and Engineering, Rensselaer Polytechnic Institute, 110 8th Street, Troy, New York 12180, USA*
[6] *Department of Applied Physical Sciences, University of North Carolina, Chapel Hill, NC 27514, North Carolina, United States*
[7] *Department of Materials Science and Engineering, University of Wisconsin-Madison, WI, 53706, USA*

(Dated: October 10, 2023)

SI. SPIN LIFETIME: SPIN RELAXATION AND DEPHASING

Spin lifetime τ_s is calculated based on the method developed in Ref. 1. To define τ_s, we follow the time evolution of the total spin observable $S_i^{\mathrm{tot}}(t)$ and the excess spin observable $\delta S_i^{\mathrm{tot}}(t)$

$$S_i^{\mathrm{tot}}(t) = \mathrm{Tr}\left(s_i(t)\,\rho(t)\right), \tag{S1}$$

$$\delta S_i^{\mathrm{tot}}(t) = S_i^{\mathrm{tot}}(t_0) - S_i^{\mathrm{tot,eq}}, \tag{S2}$$

where $S_i^{\mathrm{tot}}(t)$ is the i-component of the total spin observable vector $\mathbf{S}^{\mathrm{tot}}(t)$; $\rho(t)$ is the density matrix; s_i is spin Pauli matrix in Blöch basis along direction i; "eq" corresponds to the final equilibrium state. The time evolution must start at an initial state (at $t = t_0$) with a net spin i.e. $\delta\rho(t_0) = \rho(t_0) - \rho^{\mathrm{eq}} \neq 0$ such that $\delta S_i^{\mathrm{tot}}(t_0) \neq 0$. We evolve the density matrix through the quantum master equation given in Ref. 1 (Eq. 5 therein) for a long enough simulation time, typically from ns to μs, until the evolution of $\delta S_i^{\mathrm{tot}}(t)$ can be reliably fitted by

$$\delta S_i^{\mathrm{tot}}(t) = \delta S_i^{\mathrm{tot}}(t_0) exp\left[-\frac{t-t_0}{\tau_{s,i}}\right] \times \cos\left[\Omega\left(t-t_0\right)+\phi\right] \tag{S3}$$

to extract the spin lifetime, $\tau_{s,i}$. Above, Ω is oscillation frequency due to energy splitting in general, which under $\mathbf{B}^{\mathrm{ext}} \neq 0$ would have a magnitude of about $\mu_B B^{\mathrm{ext}} \overline{\overline{g}}$, where $\overline{\overline{g}}$ is the weighted averaged g-factor defined in Eq. 10 in the main text.

In Ref. 1, we have shown that it is suitable to generate the initial spin imbalance by applying a test magnetic field at $t = -\infty$, allowing the system to equilibrate with a net spin and then turning it off suddenly at t_0.

Historically, two types of τ_s - spin relaxation time (or longitudinal time) T_1 and ensemble spin dephasing time (or transverse time) T_2^* were used to characterize the decay of spin ensemble or $\delta\mathbf{S}^{\mathrm{tot}}(t)$[2, 3]. Suppose the spins are initially polarized along an external field $\mathbf{B}^{\mathrm{ext}}$, if we examine $\delta\mathbf{S}^{\mathrm{tot}}(t)\,||\mathbf{B}^{\mathrm{ext}}$, τ_s is called T_1; if examine $\delta\mathbf{S}^{\mathrm{tot}}(t) \perp \mathbf{B}^{\mathrm{ext}}$, τ_s is called T_2^*.

The measurement of T_1 requires longitudinal $\mathbf{B}^{\mathrm{ext}}$, which are taken small but large enough to polarize nuclear spins and suppress their contribution to spin decay. At $\mathbf{B}^{\mathrm{ext}} = 0$, experimental τ_s are usually regarded as $T_2^*\left(B^{\mathrm{ext}} \rightarrow 0\right)$ for halide perovskites, because experimental $\tau_s\left(\mathbf{B}^{\mathrm{ext}} = 0\right)$ are much shorter than T_1 but comparable to T_2^* at weak transverse $\mathbf{B}^{\mathrm{ext}}$. While our theoretical $\tau_s\left(\mathbf{B}^{\mathrm{ext}} = 0\right)$ without considering nuclear spins should be regarded as T_1.

The ensemble spin dephasing rate $1/T_2^*$ consists of both reversible and irreversible parts. The reversible part may be removed by the technique of spin echo. The irreversible part is called spin dephasing rate $1/T_2$, which must be

[a] JX and KL contributed equally to this work.
[*] sundar@rpi.edu
[†] yping3@wisc.edu

smaller than $1/T_2^*$. According to Ref. 3, T_2 may be also defined using Eq. S3 without the need of spin echo but instead of $S_i^{\mathrm{tot}}(t)$, we need another quantity - the sum of individual spin amplitudes

$$S_i^{\mathrm{indiv}} = \sum_k \left| \sum_{mn} s_{i,kmn} \rho_{knm}(t) \right|. \tag{S4}$$

In the case of two Kramers degenerate bands, if we take z direction along $\mathbf{B}^{\mathrm{ext}}$, then T_1 describes the decay of the occupation differences between two bands (the diagonal matrix element of one-particle density matrix ρ), while T_2 and T_2^* describes the decay of the off-diagonal elements of ρ.

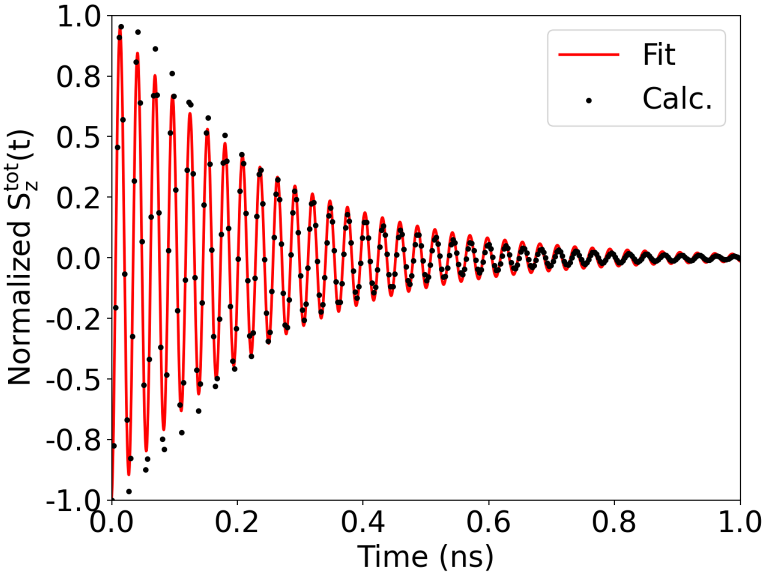

FIG. S1. Time evolution of S_z^{tot} of pristine $CsPbBr_3$ at 4 K under a transverse magnetic field of 1 Tesla $n_c = 10^{18}$ cm^{-3}, after the initial spin imbalance generated by a test magnetic field. “Calc.” denotes calculated S_z^{tot}. “Fit” denotes fitted S_z^{tot} using Eq. S3.

In Fig. S1, we compare calculated S_z^{tot} and fitted ones using Eq. S3 of pristine $CsPbBr_3$ at 4 K under a transverse magnetic field of 1 Tesla, after the initial spin imbalance generated by a test magnetic field. We find the fitted curve matches the calculated one perfectly after 0.2 ns, which gives spin lifetime $\tau_{s,i}$ and the Larmor precession frequency Ω through Eq. S3.

SII. COMPUTATIONAL DETAILS

The ground-state electronic structure, phonon, as well as electron-phonon and electron-impurity matrix elements are firstly calculated using Density Functional Theory (DFT), with relatively coarse k and q meshes in the plane-wave DFT code JDFTx[4]. We use Perdew–Burke–Ernzerhof exchange-correlation functional[5]. The structures are fully optimized and the lattice constants are 8.237, 8.514 and 11.870 Å. The phonon calculations employ $2\times2\times1$ supercells through finite difference calculations. We use Optimized Norm-Conserving Vanderbilt (ONCV) pseudopotentials[6] with self-consistent spin-orbit coupling throughout, where we find convergence at a wavefunction kinetic energy cutoff of 48 Ry.

The e-i matrix g^i between state (k,n) and (k',n') is

$$g^i_{kn,k'n'} = \langle kn | \Delta V^i | k'n' \rangle, \tag{S5}$$

$$\Delta V^i = V^i - V^0, \tag{S6}$$

where V^i is the potential of the impurity system and V^0 is the potential of the pristine system. V^i is computed with SOC using a $2\times2\times1$ supercell with a neutral impurity. To speed up the supercell convergence, we used the potential alignment method developed in Ref. 7.

We then transform all quantities from plane wave basis to maximally localized Wannier function basis, and interpolate them to substantially finer k and q meshes[8–10]. The Wannier interpolation approach fully accounts for polar terms in the e-ph matrix elements and phonon dispersion relations, using the approach developed by Verdi and Giustino[11]. The Born effective charges and electronic dielectric constants are calculated from open-source code QuantumESPRESSO[12]. The e-e scattering matrix is computed using the same method given in Ref. 1 with the macroscopic static dielectric constant about 36 computed from density functional perturbation theory (DFPT)[13] in QuantumESPRESSO. The simulations of Born effective charge Z^*, high-frequency dielectric constant ε_∞, and low-frequency dielectric constant ε_0 employ the commonly-used method developed in Ref. 13 based on Density-Functional Perturbation Theory, implemented in QuantumESPRESSO. The fine k and q meshes are $48 \times 48 \times 32$ for simulations at 300 K and are finer at lower temperature, e.g., $180 \times 180 \times 120$ for simulations at 4 K. The computation of e-i and e-e matrix elements and the real-time dynamics simulations are done with the DMD code (Density-Matrix Dynamics), interfaced with the JDFTx-code. The energy-conservation smearing parameter σ is chosen to be comparable or smaller than $k_B T$ for each calculation.

SIII. THE BAND STRUCTURE AND PHONON DISPERSION

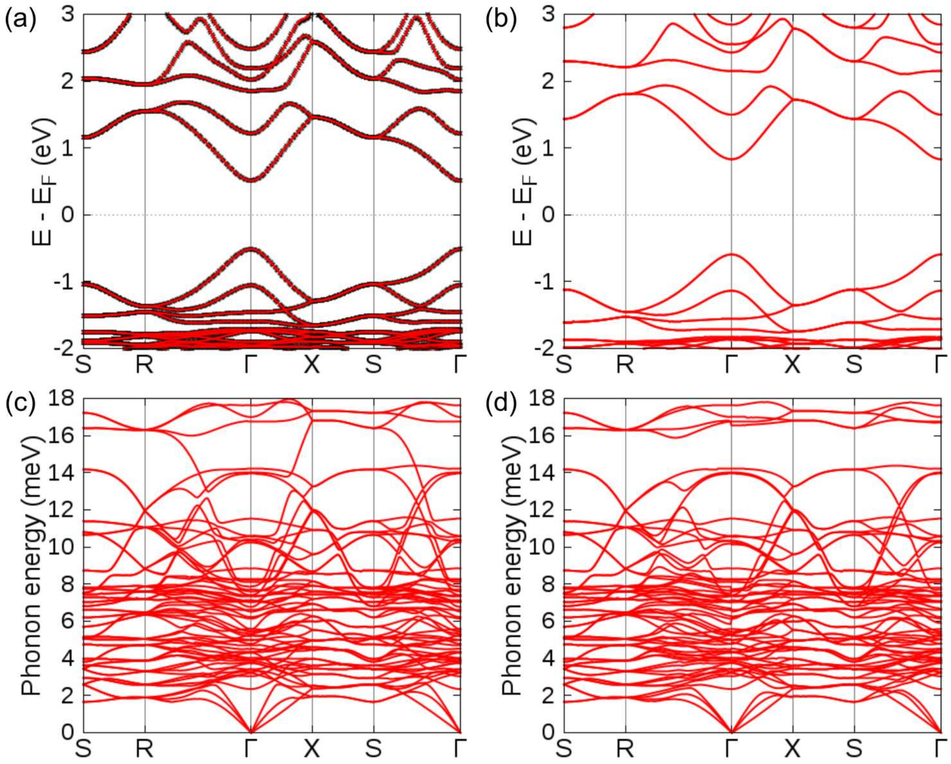

FIG. S2. (a) The band structure of $CsPbBr_3$ from DFT calculation with PBE functional and spin-orbit coupling (red) and from Wannierization (black), with the Fermi level being aligned to 0. (b) The band structure of $CsPbBr_3$ from DFT calculation with EV93PW91 functional. Phonon dispersion of $CsPbBr_3$ (c) without and (d) with considering LO-TO splitting with PBE functional. Our phonon dispersion is in good agreement with previous theoretical one reported in Ref. 14.

Figure S2a shows a direct band gap of $CsPbBr_3$ at Γ, suggesting that spin relaxation is important at Γ where carriers occupy first. The perfect overlap between the DFT band structure and Wannier band structure implies good Wannierization quality. The band structure simulated using EV93PW91 functional is shown in Fig. S2b and gives a larger band gap than PBE in Fig. S2a.

By comparing the phonon dispersion of $CsPbBr_3$ at PBE without LO-TO splitting (Fig. S2c) to that with LO-TO splitting (Fig. S2d), we found that the long-range dipole potential field strongly splits the optical modes near 14 meV at Γ. This gives rise to ∼2 meV blueshift of the No. 57 optical mode within the 60 modes in total. The No. 57 (O57) and No. 58 (O58) optical modes were found to play significant roles in carrier relaxation. The corresponding discussion can be found from section "Analysis of spin-phonon relaxation" in the main text.

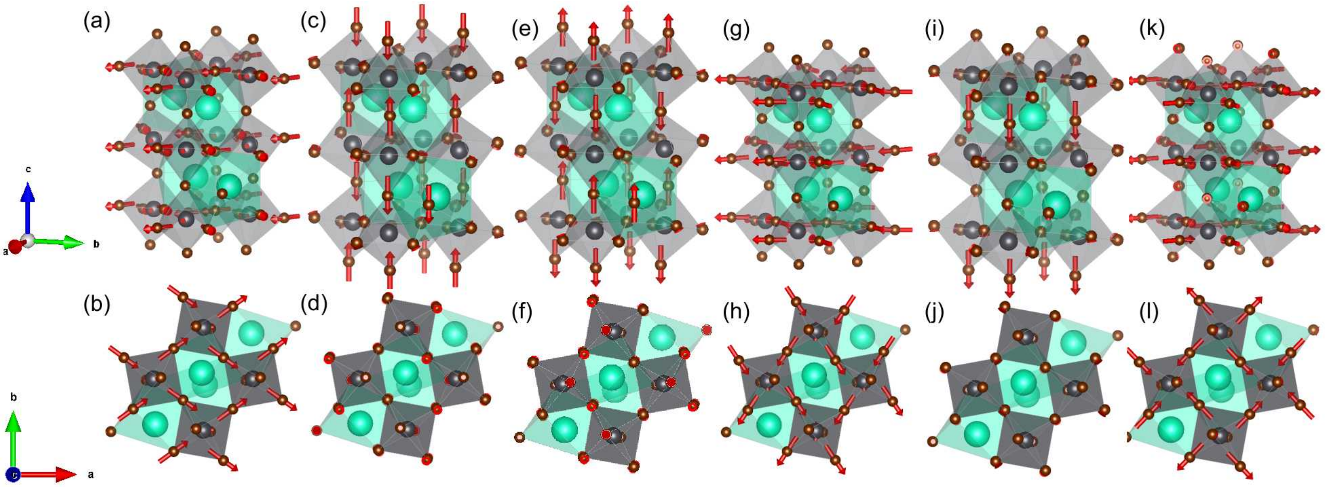

FIG. S3. Visualization of the $CsPbBr_3$ phonon modes with LO-TO splitting. Phonon modes (a)-(b) O57 and (c)-(d) O58 when $\mathbf{q} = (0.001, 0, 0)$ Bohr^{-1}, (e)-(f) O57 and (g)-(h) O58 when $\mathbf{q} = (0, 0.001, 0)$ Bohr^{-1}, and (i)-(j) O57 and (k)-(l) O58 when $\mathbf{q} = (0, 0, 0.001)$ Bohr^{-1}. The red arrows represent the phonon displacement vectors.

In terms of the symmetry, bulk $CsPbBr_3$ belongs to $Pnma$ space group (D_{2h}^{16}). By visualizing the displacement patterns as shown in Fig. S3, O57 and O58 phonon modes transform as B_{3g} and B_{2g}, respectively. And both of them are Raman-active based on symmetry.

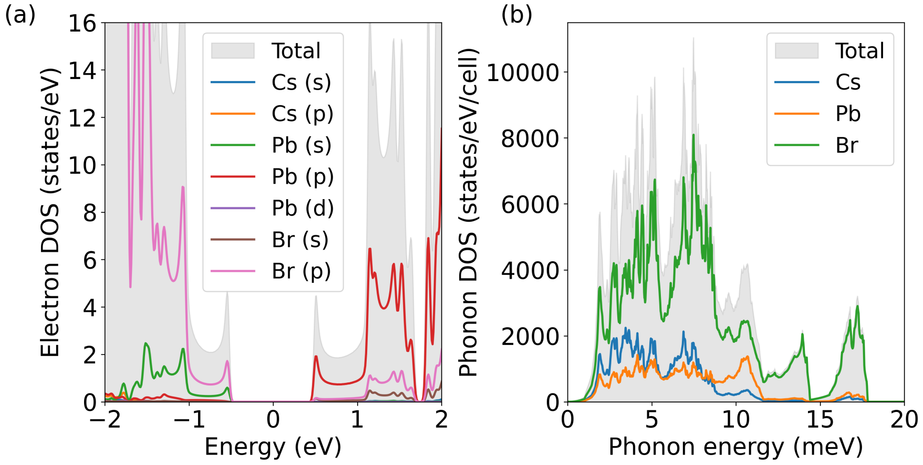

FIG. S4. (a) Electronic projected density of states (DOS) and (b) phonon projected density of states of $CsPbBr_3$.

SIV. THE BENCHMARK OF ORBITAL ANGULAR MOMENTUM L IMPLEMENTATION

To verify our implementation of orbital angular momentum, we did benchmark calculations of single-band orbital angular momentum and g-factor of A and B excitons of monolayer MoS_2. We find that our results (Table S1) are in good agreement with previous theoretical and experimental results[15–17].

	This work	Theory 1	Theory 2	Exp. 1
$L_{z,K,v-1}$	4.09	3.72	3.94	
$L_{z,K,v-1}$	4.30	3.93	4.10	
$L_{z,K,c}$	2.06	2.09	1.98	
$L_{z,K,c+1}$	1.84	1.87	1.76	
g_A	-4.48	-3.68	-4.24	-4.6
g_B	-4.50	-3.70	-4.36	-4.3

TABLE S1. The benchmark of orbital angular momentum and g factors of monolayer MoS_2. $L_{z,K,n}$ is the single-band orbital angular momentum along z direction of band n at high-symmetry **k**-point K, which is the diagonal element of $\mathbf{L}_{z,K}$ matrix. v and c denote the highest valence and lowest conduction bands respectively. g_A and g_B are g-factor of A and B excitons respectively. The theoretical g_A and g_B shown here are computed without considering excitonic effects and $g_A = 2\left(L_{K,c+1} - L_{K,v}\right)$ and $g_B = 2\left(L_{K,c} - L_{K,v-1}\right)$ (see Ref. 15). Theory 1 and 2 are theoretical results from Ref. 15 and 16 respectively. Exp. 1 are experimental data from Ref. 17.

SV. $V_{\rm xc}$-DEPENDENCE OF g-FACTOR AND ANOTHER g-FACTOR DEFINITION

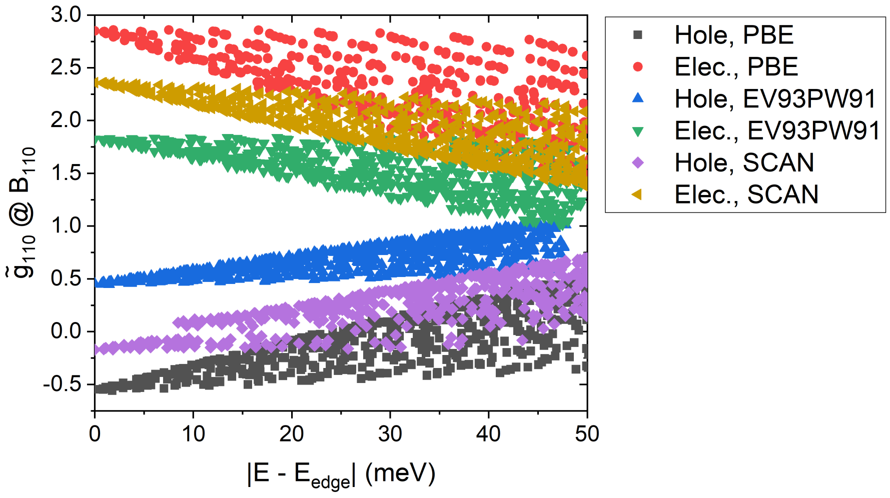

FIG. S5. The **k**-dependent g-factor $\widetilde{g}_k$ for magnetic fields along [110] direction (Eq. 8 and 9 in Method Section of the main text) computed using different exchange-correlation functionals ($V_{\rm xc}$) at **k**-points around the band edges. Each data point corresponds to a **k** point. The functional EV93PW91 uses the Engel–Vosko exchange functional[18] and the correlation part of PW91 GGA functional. The SCAN or "strongly constrained and appropriately normed" functional is a meta-GGA functional developed by Sun et al.[19]

The accurate prediction of g-factor requires accurate electronic structure as inputs, therefore we examine g-factors using DFT states from different exchange-correlation functionals ($V_{\rm xc}$). In Fig. S5, we show **k**-dependent g-factor $\widetilde{g}_k$ calculated using three different $V_{\rm xc}$ - PBE, SCAN and EV93PW91. PBE as a GGA functional and SCAN as a meta-GGA functional were commonly employed in the DFT calculations. EV93PW91 was known to improve band gap values compared with local or semi-local functionals[20]. We found that EV93PW91 predicted a better band gap of about 1.42 eV (see the EV93PW91 band structure in Fig. S2(b)), compared with the PBE one with a band gap of 1.03 eV in Fig. S2(a), and the SCAN one with a value of 1.18 eV (the experimental one is at 2.36 eV[21]). EV93PW91 predicted the electron effective mass of about $0.27m_e$, improved over PBE at $0.22m_e$ and SCAN at $0.24m_e$ respectively, against the experimental one at $0.26m_e$[14].

From Fig. S5, we find that for all $V_{\rm xc}$, the calculated electron $\widetilde{g}_k$ are larger than hole $\widetilde{g}_k$ and the sums of electron and hole $\widetilde{g}_k$ range from 1.85 to 2.4, in agreement with experiments[22, 23]. More importantly, for all $V_{\rm xc}$, $\widetilde{g}_k$ of electrons and holes decrease and increase with state energy respectively, the fluctuation of $\widetilde{g}_k$ increases with the state energy

and the g-factor fluctuation amplitudes $\Delta\widetilde{g}$ are of the same order of magnitude.

However, both electron and hole $\widetilde{g}_k$ are found sensitive to V_{xc}.; in particular, the signs of hole $\widetilde{g}_k$ are different among different V_{xc}. Overall, we find that the magnitudes of g-factors predicted by EV93PW91 are in the best agreement with experiments. EV93PW91 predicts electron g-factor ∼1.8 and hole ∼0.5 at Γ respectively, close to experimental data[23] (1.69-2.06 for electrons and 0.65-0.85 for holes). On the other hand, both PBE[5] and SCAN[19] functionals overestimate electron g-factor and underestimate hole g-factor compared with experimental values[22, 23]. Furthermore, the anisotropy of hole g-factor along three crystal directions is found greater than that of electron, in agreement with experiments[23]. With EV93PW91, the theoretical anisotropy ratio P of electron and hole g-factors at Γ are 6% and 15% respectively, in reasonable agreement with experiments (10% for e and 13% for h)[23], where $P = |g_{max} - g_{min}|/|g_{max} + g_{min}|$ with g_{max} (g_{min}) the maximum (minimum) value of g-factors among three directions.

The strong V_{xc}-dependence of g-factors indicate that accurate electronic structure is important for quantitative comparison of g-factor with experiments. Therefore, to reliably predict the g-factor values, we may need to employ a higher level of theory, such as the GW approximation[16, 24], to improve the electronic structure description and lessen such dependence on the choice of DFT V_{xc}. On the other hand, T_2^* only depends on $\Delta\widetilde{g}$, which are less sensitive to V_{xc}, e.g., $\Delta\widetilde{g}$ by the SCAN functional[19] is ∼80% of that by PBE. Moreover, the trends of g-factors and $\Delta\widetilde{g}$ versus electronic energies are the same for different V_{xc}. Therefore, we expect that different V_{xc} predict similar magnitudes of T_2^* and the same trends of T_2^* versus external conditions.

Below, we provide a more generate definition of g-factor and its fluctuation amplitude, which is more appropriate when spin directions (at different $\mathbf{k}$) are not parallel to the direction of the applied $\mathbf{B}^{\mathrm{ext}}$, and materials are highly anisotropic.

Generally speaking, except at some high-symmetry $\mathbf{k}$-points, $\mathbf{L}$ and $\mathbf{L} + g_0\mathbf{S}$ may not be proportional to $\mathbf{S}$. Since under finite $\mathbf{B}^{\mathrm{ext}}$, the expectation value vectors of $\mathbf{L} + g_0\mathbf{S}$ must be parallel to $\mathbf{B}^{\mathrm{ext}}$ (in the first-order perturbation level), spin expectation value vectors may not be parallel to $\mathbf{B}^{\mathrm{ext}}$. Therefore, it is helpful to define a vector of Larmor precession frequency whose magnitude is equal to the energy splitting,

$$\overrightarrow{\Omega}_k\left(\mathbf{B}^{\mathrm{ext}}\right) = \Delta E_k\left(\mathbf{B}^{\mathrm{ext}}\right)\widehat{S}_{kh}^{\mathrm{exp}}\left(\widehat{\mathbf{B}^{\mathrm{ext}}}\right), \tag{S7}$$

$$\widehat{S}_{k,h}^{\mathrm{exp}}\left(\widehat{\mathbf{B}^{\mathrm{ext}}}\right) = \overrightarrow{S}_{k,h}^{\mathrm{exp}}\left(\widehat{\mathbf{B}^{\mathrm{ext}}}\right) / \left|\overrightarrow{S}_{k,h}^{\mathrm{exp}}\left(\widehat{\mathbf{B}^{\mathrm{ext}}}\right)\right|, \tag{S8}$$

where $\overrightarrow{S}_{kh}^{\mathrm{exp}}\left(\widehat{\mathbf{B}^{\mathrm{ext}}}\right)$ is the spin expectation value vector.

With the distribution of $\overrightarrow{\Omega}_k$, we can define a g-factor vector and a more appropriate g-factor fluctuation amplitude for spin dephasing.

With $\overrightarrow{\Omega}_k$, we can define a g-factor vector (with $C^{S\rightarrow J}$ defined in the main text) as

$$\overrightarrow{g}_k^{\Omega}\left(\widehat{\mathbf{B}^{\mathrm{ext}}}\right) = C^{S\rightarrow J}\frac{\overrightarrow{\Omega}_k\left(\mathbf{B}^{\mathrm{ext}}\right)}{\mu_B B^{\mathrm{ext}}}. \tag{S9}$$

With $\overrightarrow{g}_k^{\Omega}(\widehat{x})$, $\overrightarrow{g}_k^{\Omega}(\widehat{y})$ and $\overrightarrow{g}_k^{\Omega}(\widehat{z})$, we will obtain a g-factor tensor.

A more appropriate definition of g-factor fluctuation amplitude for spin dephasing requires the detailed knowledge of $\overrightarrow{\Omega}_k$. Suppose the total excited or excess spin $\delta\mathbf{S}^{\mathrm{tot}}$ is perpendicular to $\mathbf{B}^{\mathrm{ext}}$, i.e., $\delta\mathbf{S}^{\mathrm{tot}} \perp \widehat{\mathbf{B}^{\mathrm{ext}}}$, then without considering the EY spin relaxation, τ_s will be mainly determined by $\Delta\Omega_{\perp\delta\mathbf{S}^{\mathrm{tot}}}$ - the fluctuation amplitude of $\overrightarrow{\Omega}_{\perp\delta\mathbf{S}^{\mathrm{tot}}}$, which is the component of $\overrightarrow{\Omega}$ perpendicular to $\delta\mathbf{S}^{\mathrm{tot}}$.[2, 25] Suppose the unit vectors $\widehat{e}_1$, $\widehat{e}_2$ and $\widehat{e}_3$ orthogonal among each other, then similar to Eq. 11 in the main text, we can define $\Delta\Omega_{\perp\widehat{e}_1}$ as

$$\Delta\Omega_{\perp\widehat{e}_1} = \sqrt{\left(\Delta\Omega_{\widehat{e}_2}\right)^2 + \left(\Delta\Omega_{\widehat{e}_3}\right)^2}, \tag{S10}$$

$$\Delta\Omega_{\widehat{e}} = \sqrt{\frac{\sum_k\left(-f_k'\right)\left|\Omega_{k,\widehat{e}} - \overline{\Omega_{\widehat{e}}}\right|^2}{\sum_k\left(-f_k'\right)}}, \tag{S11}$$

$$\overline{\Omega_{\widehat{e}}} = \frac{\sum_k\left(-f_k'\right)\Omega_{k,\widehat{e}}}{\sum_k\left(-f_k'\right)}, \tag{S12}$$

where $\Omega_{\widehat{e}}$ is the $\widehat{e}$ component of $\overrightarrow{\Omega}$. As $\delta\mathbf{S}^{\mathrm{tot}}$ is approximated rotating about $\widehat{\mathbf{B}^{\mathrm{ext}}}$, $\overrightarrow{\Omega}_{\perp\delta\mathbf{S}^{\mathrm{tot}}}$ changes with time. Suppose the unit vectors $\widehat{e}_a$ and $\widehat{e}_b$ satisfy $\widehat{e}_a \perp \widehat{e}_b$, $\widehat{e}_a \perp \widehat{\mathbf{B}^{\mathrm{ext}}}$ and $\widehat{e}_b \perp \widehat{\mathbf{B}^{\mathrm{ext}}}$, we can define an effective fluctuation

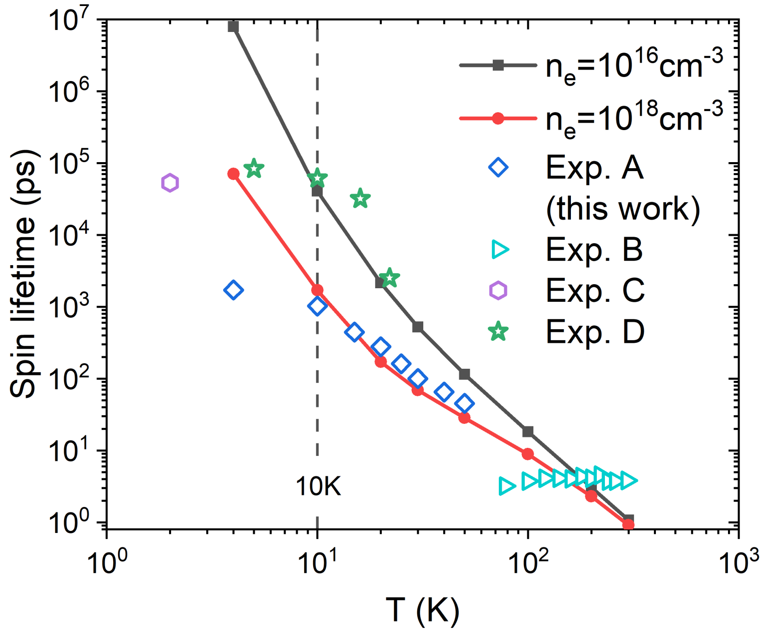

FIG. S6. Spin lifetime τ_s of $CsPbBr_3$ electrons due to both e-ph and e-e scatterings calculated as a function of T at different electron densities n_e compared with experimental data. The data points are the same as those in Fig. 1a in the main text but here we use log-scale for both y- and x-axes to highlight low-T region. The meanings of Exp. A, B, C and D are the same as in Fig. 1a in the main text.

amplitude of $\overrightarrow{\Omega}\left(\mathbf{B}^{\text{ext}}\right)$ as

$$\Delta\Omega\left(\mathbf{B}^{\text{ext}}\right) = \sqrt{\frac{\left(\Delta\Omega_{\perp\widehat{e}_a}\right)^2 + \left(\Delta\Omega_{\perp\widehat{e}_b}\right)^2}{2}} = \sqrt{\left(\Delta\Omega_{\widehat{\mathbf{B}^{\text{ext}}}}\right)^2 + \frac{1}{2}\left(\Delta\Omega_{\widehat{e}_a}\right)^2 + \frac{1}{2}\left(\Delta\Omega_{\widehat{e}_b}\right)^2}. \tag{S13}$$

With $\Delta\Omega\left(\mathbf{B}^{\text{ext}}\right)$, we can define a T and μ_c dependent effective fluctuation amplitude of g-factor under $\mathbf{B}^{\text{ext}}$,

$$\Delta g^{\Omega}\left(\widehat{\mathbf{B}^{\text{ext}}}\right) = \frac{\Delta\Omega\left(\mathbf{B}^{\text{ext}}\right)}{\mu_B B^{\text{ext}}}. \tag{S14}$$

For $CsPbBr_3$, we find Eq. S14 predicts quite similar values to those by Eq. 11 in the main text (differences are not greater than 10%).

SVI. SPIN RELAXATION TIMES

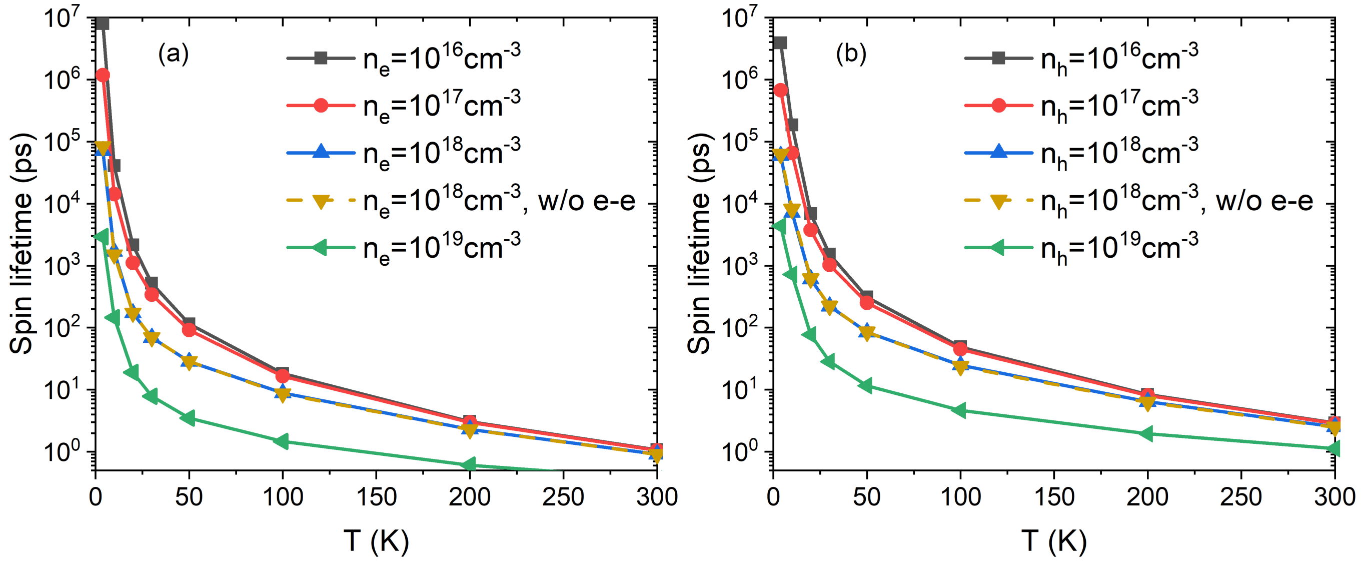

FIG. S7. (a) Electron and (b) hole τ_s of pristine $CsPbBr_3$ as a function of T at different carrier density n_c including both electron-phonon and electron-electron scatterings. The brown triangle lines represent τ_s without electron-electron (w/o e-e) scatterings.

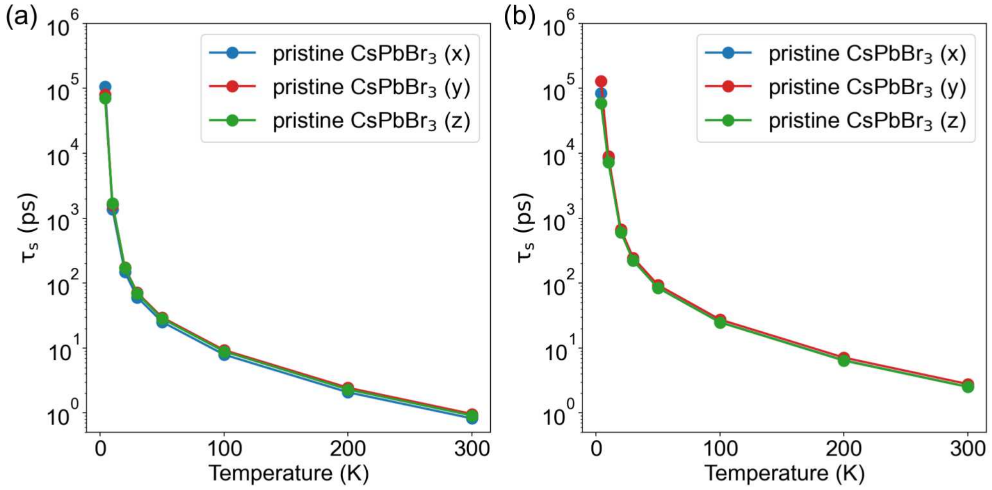

FIG. S8. (a) Electron and (b) hole τ_s of pristine $CsPbBr_3$ along x, y and z Cartesian directions with carrier density $n_c = 10^{18}$ cm^{-3} as a function of temperature.

In Fig. S8, we show electron and hole τ_s of pristine $CsPbBr_3$ along x, y and z directions and we find both of them are nearly isotropic.

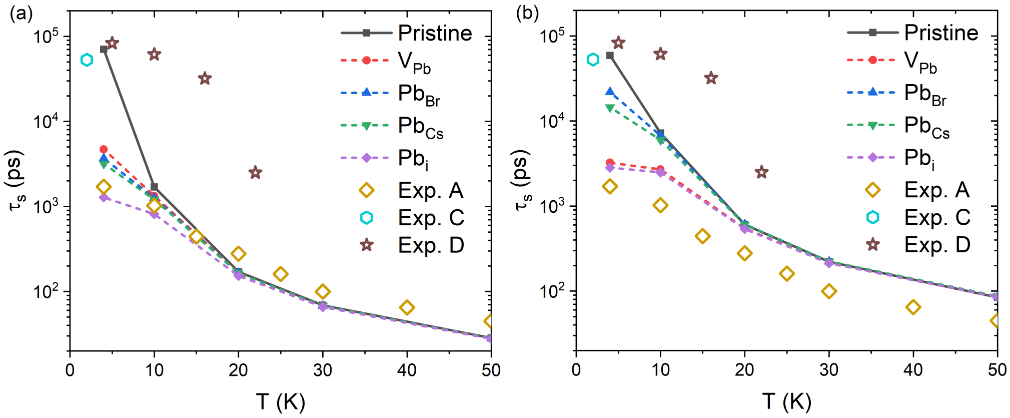

FIG. S9. Spin lifetime τ_s of (a) electrons and (b) holes in $CsPbBr_3$ calculated with and without neutral impurities at density of 10^{18} cm^{-3} compared with experiments. Here carrier density n_c is at 10^{18} cm^{-3}. V_{Pb} denotes Pb vacancy; Pb_{Br} and Pb_{Cs} denote Pb substitution of Br or Cs atoms; Pb_i denotes an extra Pb atom at an interstitial site.

Fig. S9 shows the effects of impurity scattering on τ_s at $\mathbf{B}^{ext} = 0$ as a function of T, with four representative Pb-related defects/impurities (see the results of other impurities below in Fig. S10). We found that even with a high impurity density n_i=10^{18} cm^{-3}, which is within the experimental range of 10^{14}~$10^{20}cm^{-3}$[26–28], impurity effects are negligible at $T \geqslant$20 K. At lower T, however the presence of impurities reduces τ_s, consistent with EY mechanism, and leads to a weaker T-dependence of τ_s (as the e-i scattering is T-independent). Moreover, we found that the contribution of e-i scatterings depends on the specific chemical composition of impurity, and the same defect affects differently for the electron and hole τ_s (Fig. S9). Overall, we emphasize that the quantitative description of impurity effect requires explicit atomistic simulations of impurities, given the large variation among them. They are only important at relatively low temperature T <20 K, with relatively high n_i (e.g., >10^{18} cm^{-3}).

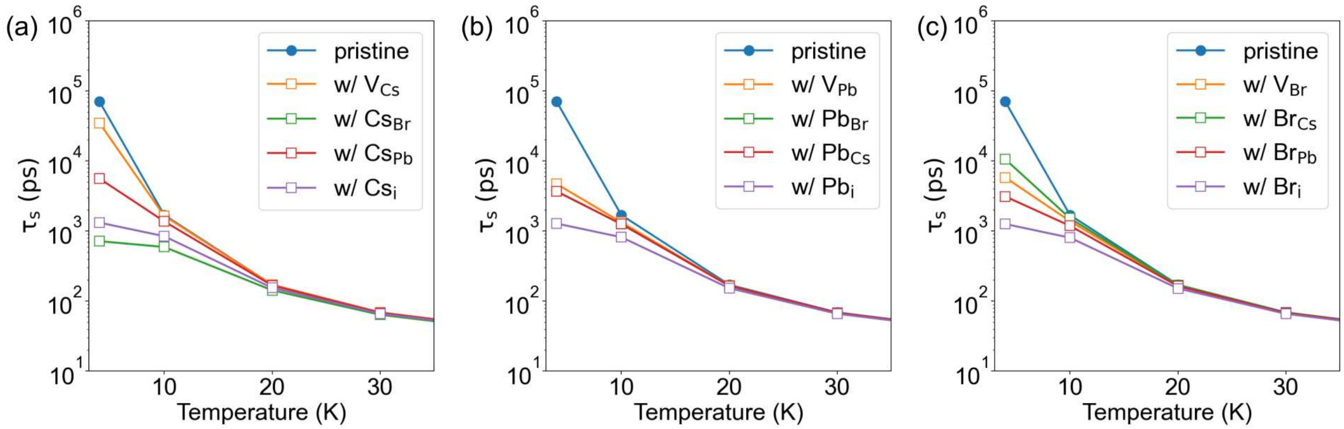

FIG. S10. Electron τ_s of $CsPbBr_3$ with different types of point defects/impurities. Both electron carrier density n_e and impurity density n_i are 10^{18} cm^{-3}. (a) With neutral Cs-derived impurities, where V_{Cs} denotes Cs vacancy; Cs_{Br} denotes Cs substitution of Br; Cs_{Pb} denotes Cs substitution of Pb; Cs_i denotes interstitial Cs doping. (b) With neutral Pb-derived impurities. (c) With neutral Br-derived impurities.

From Fig. S10, we find that the impurity effects are sensitive to the atomistic details of impurities, but all impurities studied here cannot affect τ_s at $T \geqslant$20 K if impurity density n_i is not extremely high (e.g. < 10^{18} cm^{-3}).

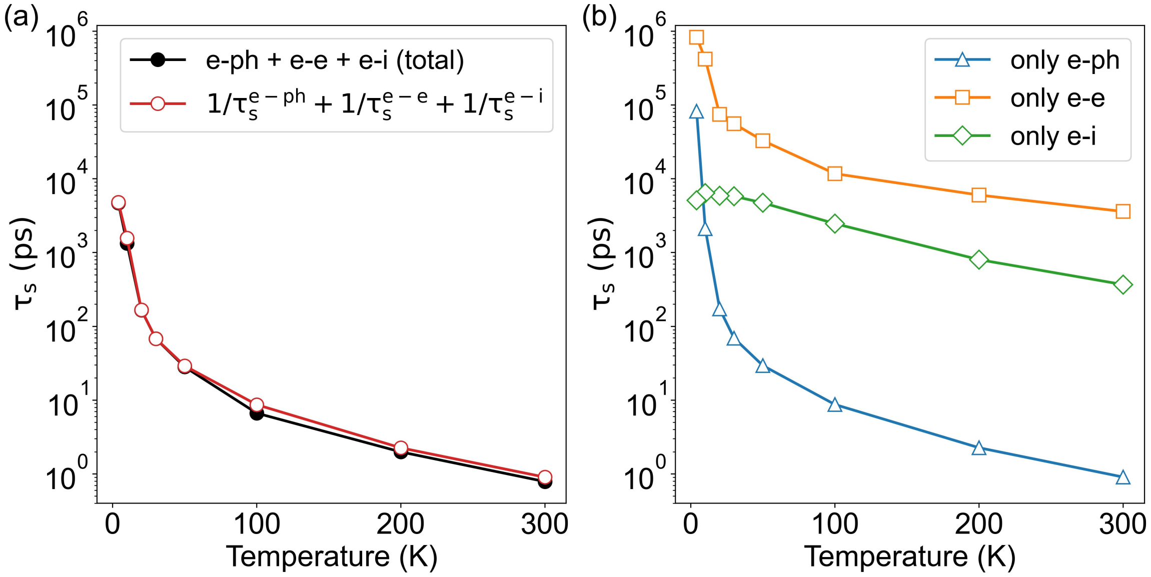

FIG. S11. (a) Electron τ_s of $CsPbBr_3$ from real-time dynamics including all of the electron-phonon (e-ph), electron-electron (e-e) and electron-impurity (e-i) scatterings (black line), and that evaluated by using the equation $1/\tau_s = 1/\tau_s^{e-ph} + 1/\tau_s^{e-e} + 1/\tau_s^{e-i}$. (b) Electron τ_s of $CsPbBr_3$ due to each of the e-ph, e-e and e-i scatterings. Both electron carrier density n_e and impurity density n_i are 10^{18} cm^{-3}. Impurity V_{Pb} is considered in the e-i scattering.

Fig. S11(a) shows that the total τ_s decreases when the scatterings are stronger (higher temperature and adding e-i scattering), indicating that EY mechanism is the major mechanism of bulk $CsPbBr_3$ (in absence of external B field). Furthermore, τ_s evaluated by using the equation $1/\tau_s = 1/\tau_s^{e-ph} + 1/\tau_s^{e-e} + 1/\tau_s^{e-i}$ is nearly the same as that from the real-time dynamics simulation including all the scatterings. In this circumstance, we can separate the causes of spin relaxation into e-ph, e-e, and e-i scatterings as shown in Fig. S11(b). τ_s due to e-e scattering is the longest compared to those due to e-ph and e-i scatterings, so that it can be ignored. At T > 10K, the e-ph scattering is the strongest scattering channel because the excitation of phonons is considerable, as a result, τ_s due to the e-ph scattering is the shortest. When T = 4K, there are less phonons excited, so that with a high impurity density n_i, the e-i scattering dominates spin relaxation. τ_s^{e-i} is weakly temperature-dependent. This weak dependence is due to temperature broadening effects and the temperature dependence of the chemical potential with a fixed carrier density.

SVII. CARRIER AND SPIN TRANSPORT PROPERTIES IN LOW DENSITY LIMIT

We calculate the electron mobility μ_e and the hole mobility μ_h by solving the linearized Boltzmann equation in relaxation-time approximation[29–32],

$$\mu_{e(h),i} = \frac{e}{n_{e(h)} V N_k} \sum_{k,n\in \mathrm{CB(VB)}} \frac{df}{d\epsilon}|_{\epsilon=\mu_F} v_{kn,i}^2 \tau_{m,kn}, \tag{S15}$$

where $i = x, y, z$ for three dimensional systems. N_k is the number of k points. V is the unit cell volume. n_e and n_h are electron and hole density respectively. CB and VB denote conduction and valence bands, respectively. f is Fermi-Dirac function. μ_F is the chemical potential. v is the band velocity. τ_m is the momentum relaxation time. Using the Matthiessen's rule, we have

$$\tau_{m,kn}^{-1} = \left(\tau_{m,kn}^{\mathrm{e-ph}}\right)^{-1} + \left(\tau_{m,kn}^{\mathrm{e-i}}\right)^{-1} + \left(\tau_{m,kn}^{\mathrm{e-e}}\right)^{-1}, \tag{S16}$$

where $\tau_m^{\mathrm{e-ph}}$, $\tau_m^{\mathrm{e-i}}$ and $\tau_{m,kn}^{\mathrm{e-e}}$ are the electron-phonon, electron-impurity and electron-electron momentum relaxation times, respectively, which read[1, 29–32]

$$\left(\tau_{m,kn}^{c}\right)^{-1} = \frac{1}{N_k}\sum_{k'n'}\left(\tau_{kn\rightarrow k'n'}^{c}\right)^{-1}\left(1-\cos\theta_{k'n'kn}\right), \tag{S17}$$

$$\left(\tau_{kn\rightarrow k'n'}^{\mathrm{e-ph}}\right)^{-1} = \frac{2\pi}{\hbar}\sum_{\lambda\pm}\left|g_{k'n',kn}^{k'-k,\lambda}\right|^2\left(n_{k'-k,\lambda}+0.5\mp\left(0.5-f_{k'n'}\right)\right)\delta\left(\epsilon_{k'n'}-\epsilon_{kn}\mp\hbar\omega_{k'-k,\lambda}\right), \tag{S18}$$

$$\left(\tau_{kn\rightarrow k'n'}^{\mathrm{e-i}}\right)^{-1} = n_i V\frac{2\pi}{\hbar}\left|g_{k'n',kn}^{i}\right|^2\delta\left(\epsilon_{k'n'}-\epsilon_{kn}\right), \tag{S19}$$

$$\left(\tau_{kn\rightarrow k'n'}^{\mathrm{e-e}}\right)^{-1} = \frac{2\pi}{\hbar}\sum_{k_3n_3k_4n_4}\left\{\begin{array}{c}\left|g_{kn,k_3n_3,k'n',k_4n_4}\right|^2\delta_{k+k_3-k'-k_4}\\ \times\left[\begin{array}{c}f_{k'n'}^{\mathrm{eq}}f_{k_4n_4}^{\mathrm{eq}}\left(1-f_{k_3n_3}^{\mathrm{eq}}\right)+\\ \left(1-f_{k'n'}^{\mathrm{eq}}\right)f_{k_3n_3}^{\mathrm{eq}}\left(1-f_{k_4n_4}^{\mathrm{eq}}\right)\end{array}\right]\\ \times\delta\left(\epsilon_{kn}+\epsilon_{k_3n_3}-\epsilon_{k'n'}-\epsilon_{k_4n_4}\right)\end{array}\right\} \tag{S20}$$

$$\cos\theta_{k'n'kn} = \frac{\overrightarrow{v}_{k'n'}\cdot\overrightarrow{v}_{kn}}{|\overrightarrow{v}_{k'n'}||\overrightarrow{v}_{kn}|}, \tag{S21}$$

where c represents $e-e$, $e-ph$, or $e-e$; $g_{k'n',kn}^{k'-k,\lambda}$ is the e-ph matrix element between state (k',n') and state (k,n) with phonon mode λ; and $g_{k'n',kn}^{i}$ is the e-i matrix element defined in Eq. S5 and computed with DFT supercells with neutral impurities. $n_{k'-k,\lambda}$ is the phonon occupation number. $g_{k_1n_1,k_3n_3,k_2n_2,k_4n_4}$ is the e-e matrix element defined in Eq. A6 in Ref. 1. f_{kn}^{eq} is the equilibrium occupation of electronic state (k,n).

We compute spin diffusion length l_s for z-direction spin transport and spin polarization using the relation[25] $l_s = \sqrt{D\tau_s}$, where D is diffusion coefficient. D can be estimated using the general form of Einstein relation[33] $D = \mu_c n_c / \frac{dn_c}{d\mu_{F,c}}$, where μ_c is the free-carrier mobility, $\mu_{F,c}$ is the chemical potential, and n_c is the carrier density.

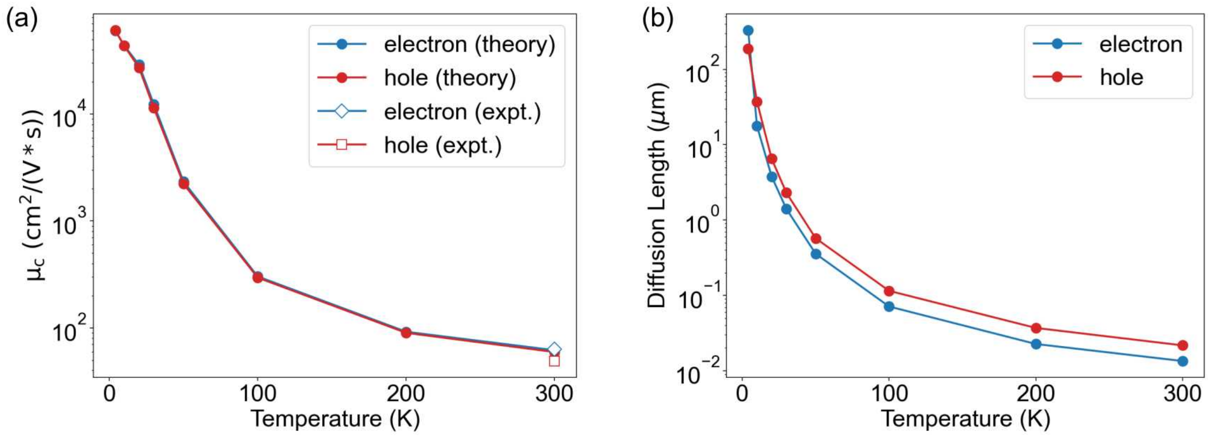

FIG. S12. Calculated mobility μ_c (a) and spin diffusion length l_s (b) of electrons of pristine $CsPbBr_3$ in low density limit as a function of temperature. "expt." denotes experimental data from Ref. [34].

Fig. S12 shows calculated mobility μ_c and spin diffusion length l_s of electrons of pristine $CsPbBr_3$ in low density limit (here n_c is taken as 10^{14} cm^{-3}), which set the upper bounds of μ_c and l_s. Considering that there are no impurities and the e-e scattering is not active in low density limit, only the e-ph scattering contributes here. From Fig. S12a and b, we find that both μ_c and l_s increase fast with decreasing T and can reach very high values at low T, e.g., l_s can be as long as hundreds of μm at 4 K.

SVIII. MAGNETIC-FIELD EFFECTS ON τ_s

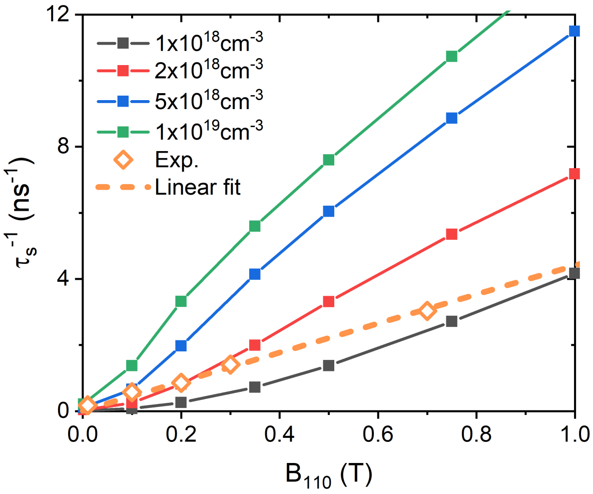

FIG. S13. The effects of transverse magnetic fields (perpendicular to spin direction) on electron τ_s of pristine $CsPbBr_3$ under $B \leq$1 Tesla. Different solid lines denote τ_s at different electron carrier density. The estimated experimental carrier density is around 10^{18}cm^{-3} (corresponding to the black line here). The orange empty diamond denotes the experimental values, with dashed line as their linearly fitted values.

From Fig. S13, we find that the calculated $\tau_s^{-1}\left(\mathbf{B}^{\text{ext}}\right)$ is proportional to $\left(B^{\text{ext}}\right)^2$ at low B^{ext} (details in SI Fig. S13) following the DP mechanism.

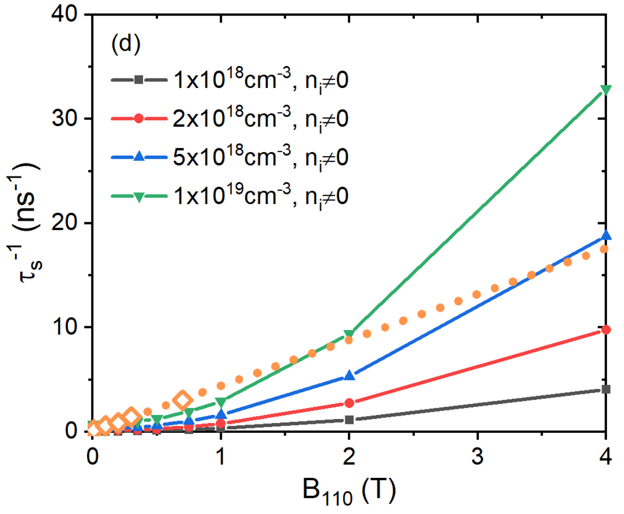

FIG. S14. τ_s^{-1} as a function of B^{ext} at 4 K at different n_e considering the e-i scattering with 10^{17} cm^{-3} V_{Pb} neutral impurities.

By comparing Fig. 5c in the main text and Fig. S14d, we conclude that introducing more scattering such as adding impurities, weakens the $\mathbf{B}^{\text{ext}}$-dependence (τ_s^{-1} increases slower with $\mathbf{B}^{\text{ext}}$). The explanation is as follows. More scatterings lead to smaller τ_p (thus smaller $\tau_p\Delta\Omega$, $(\tau_s^{\Delta\Omega})^{-1}$ closer to strong scattering limit in regime (ii), dominated by DP mechanism $\left(\tau_s^{\text{DP}}\right)^{-1}$). The latter is often much smaller than FID rate $\left(\tau_s^{\text{FID}}\right)^{-1}$ in regime (i) (the weak scattering limit). Meanwhile, more impurity scatterings give large zero-$\mathbf{B}$-field rate $\left(\tau_s^0\right)^{-1}$. Together, increasing external scatterings, leading to an increase of $\left(\tau_s^0\right)^{-1}$ and a decrease of $\left(\tau_s^{\Delta\Omega}\right)^{-1}$, finally weakens the $\mathbf{B}^{\text{ext}}$-dependence of $\tau_s^{-1}\left(\mathbf{B}^{\text{ext}}\right)$. From Fig. S14d, we find that with relatively strong impurity scattering (e.g, with 10^{17} cm^{-3} V_{Pb} neutral impurities), the $\mathbf{B}^{\text{ext}}$-dependence of τ_s is in disagreement with experiments, indicating that impurity scattering is probably weaker in those experiments.

	Holes	Electrons
Relevant isotope	^{207}Pb	^{79}Br and ^{81}Br
Number of relevant nuclei in unitcell	4	12
Nulcear spin I	1/2	3/2
Abundance α	22.1%	Totally 100%
Hyperfine constant A (μeV)	∼25	∼1.75
Unit-cell volume V_u (nm^3)	0.833	
C^{loc} (nm^3/ns^2, main-text Eq. 31)	∼530	∼180
Localization radii (nm)	2.5-14 (Ref. 35–38)	
$T^*_{2,\mathrm{loc}}$ (ns)	0.35-4.6	0.6-8.0

TABLE S2. Parameters used to estimate emsemble spin dephasing time of localized carriers $T^*_{2,\mathrm{loc}}$ of orthorhombic $CsPbBr_3$ due to nuclear spin fluctuation. We consider the Fermi contact contribution to hyperfine coupling, which was assumed to be the most important contribution in Refs. 22, 39, 40 for $CsPbBr_3$ and GaAs. For the Fermi contact contribution, s orbital is relevant since its wavefunction is considerable at the positions of the nuclei, while p and d orbitals are irrelevant. Considering that s orbitals of Pb and Br contribute considerably to Bloch functions of holes and electrons respectively, ^{207}Pb and $^{79/81}$Br with non-zero I are relevant isotopes to hyperfine coupling for holes and electrons respectively. According to Eq. 27 in the main text, $A \propto 1/V_u$, A of orthorhombic $CsPbBr_3$ is approximately 1/4 of A of cubic $CsPbBr_3$, considering that their Bloch functions at the band edge are similar (e.g., their hole Bloch functions are both s-orbital-like) and V_u of orthorhombic $CsPbBr_3$ is about 4 times of that of cubic $CsPbBr_3$.

SIX. THE C-PPR(T) MEASUREMENTS

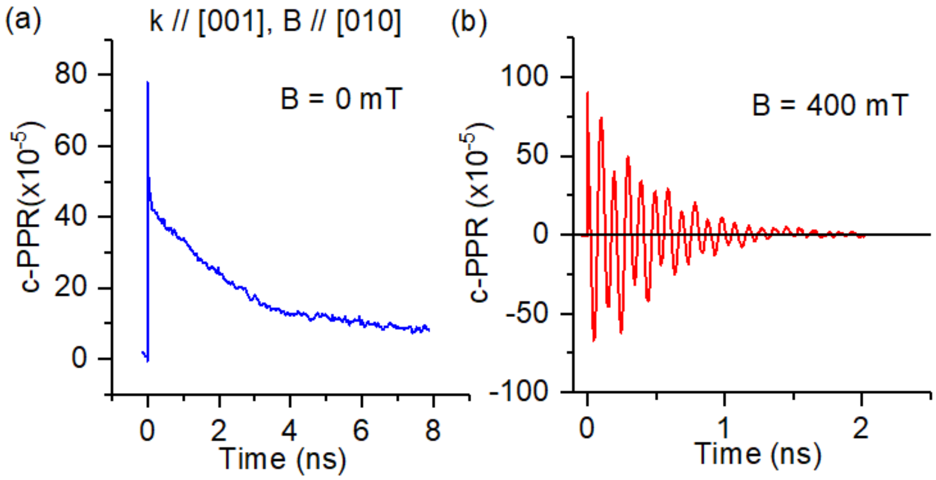

FIG. S15. Transient circularly-polarized photoinduced reflection in $CsPbBr_3$ single crystal excited at 405 nm measured at 4K on the (001) facet with B along [010] orientation. (a) B=0 mT and (b) B=400 mT. The spin lifetime in (a) is measured after the 'coherence artefact' seen at t=0. The spin lifetime in (b) is measured from the decay of the quantum beatings of the photocarriers.

[1] J. Xu, A. Habib, R. Sundararaman, and Y. Ping, Phys. Rev. B **104**, 184418 (2021).
[2] M. Wu, J. Jiang, and M. Weng, Phys. Rep. **493**, 61 (2010).
[3] C. Lü, J. Cheng, M. Wu, and I. da Cunha Lima, Phys. Lett. A **365**, 501 (2007).

[4] R. Sundararaman, K. Letchworth-Weaver, K. A. Schwarz, D. Gunceler, Y. Ozhabes, and T. A. Arias, SoftwareX **6**, 278 (2017).
[5] J. P. Perdew, K. Burke, and M. Ernzerhof, Phys. Rev. Lett. **77**, 3865 (1996).
[6] D. R. Hamann, Phys. Rev. B **88**, 085117 (2013).
[7] R. Sundararaman and Y. Ping, J. Chem. Phys. **146**, 104109 (2017).
[8] N. Marzari and D. Vanderbilt, Phys. Rev. B **56**, 12847 (1997).
[9] A. M. Brown, R. Sundararaman, P. Narang, W. A. Goddard, and H. A. Atwater, ACS Nano **10**, 957 (2016).
[10] A. Habib, R. Florio, and R. Sundararaman, J. Opt. **20**, 064001 (2018).
[11] C. Verdi and F. Giustino, Phys. Rev. Lett. **115**, 176401 (2015).
[12] P. Giannozzi, S. Baroni, N. Bonini, M. Calandra, R. Car, C. Cavazzoni, D. Ceresoli, G. L. Chiarotti, M. Cococcioni, I. Dabo, *et al.*, J. Phys. Condens. Matter **21**, 395502 (2009).
[13] X. Gonze and C. Lee, Phys. Rev. B **55**, 10355 (1997).
[14] M. Puppin, S. Polishchuk, N. Colonna, A. Crepaldi, D. Dirin, O. Nazarenko, R. De Gennaro, G. Gatti, S. Roth, T. Barillot, *et al.*, Phys. Rev. Lett. **124**, 206402 (2020).
[15] T. Woźniak, P. E. F. Junior, G. Seifert, A. Chaves, and J. Kunstmann, Phys. Rev. B **101**, 235408 (2020).
[16] T. Deilmann, P. Krüger, and M. Rohlfing, Phys. Rev. Lett. **124**, 226402 (2020).
[17] A. Mitioglu, K. Galkowski, A. Surrente, L. Klopotowski, D. Dumcenco, A. Kis, D. K. Maude, and P. Plochocka, Phys. Rev. B **93**, 165412 (2016).
[18] E. Engel and S. H. Vosko, Phys. Rev. B **47**, 13164 (1993).
[19] J. Sun, A. Ruzsinszky, and J. P. Perdew, Phys. Rev. Lett. **115**, 036402 (2015).
[20] P. Borlido, J. Schmidt, A. W. Huran, F. Tran, M. A. Marques, and S. Botti, Npj Comput. Mater. **6**, 1 (2020).
[21] T. Paul, B. Chatterjee, S. Maiti, S. Sarkar, N. Besra, B. Das, K. Panigrahi, S. Thakur, U. Ghorai, and K. Chattopadhyay, J. Mater. Chem. C **6**, 3322 (2018).
[22] V. V. Belykh, D. R. Yakovlev, M. M. Glazov, P. S. Grigoryev, M. Hussain, J. Rautert, D. N. Dirin, M. V. Kovalenko, and M. Bayer, Nat. Commun. **10**, 673 (2019).
[23] E. Kirstein, D. Yakovlev, M. Glazov, E. Zhukov, D. Kudlacik, I. Kalitukha, V. Sapega, G. Dimitriev, M. Semina, M. Nestoklon, *et al.*, Nat. Commun. **13**, 3062 (2022).
[24] F. Xuan and S. Y. Quek, Phys. Rev. Research **2**, 033256 (2020).
[25] I. Žutić, J. Fabian, and S. D. Sarma, Rev. Mod. Phys. **76**, 323 (2004).
[26] D. Zhang, Y. Yang, Y. Bekenstein, Y. Yu, N. A. Gibson, A. B. Wong, S. W. Eaton, N. Kornienko, Q. Kong, M. Lai, *et al.*, J. Am. Chem. Soc. **138**, 7236 (2016).
[27] Y. Zhu, Q. Cui, J. Chen, F. Chen, Z. Shi, X. Zhao, and C. Xu, ACS Appl. Mater. Interfaces **13**, 6820 (2021).
[28] B. A. Koscher, Z. Nett, and A. P. Alivisatos, ACS Nano **13**, 11825 (2019).
[29] S. Poncé, W. Li, S. Reichardt, and F. Giustino, Rep. Prog. Phys. **83**, 036501 (2020).
[30] C. J. Ciccarino, T. Christensen, R. Sundararaman, and P. Narang, Nano Lett. **18**, 5709 (2018).
[31] T. Gunst, T. Markussen, K. Stokbro, and M. Brandbyge, Phys. Rev. B **93**, 035414 (2016).
[32] G. D. Mahan, *Many-Particle Physics* (Springer US, 2000).
[33] R. Kubo, Rep. Prog. Phys. **29**, 255 (1966).
[34] Y. He, Z. Liu, K. M. McCall, W. Lin, D. Y. Chung, B. W. Wessels, and M. G. Kanatzidis, Nucl. Instrum. Methods Phys. Res. A **922**, 217 (2019).
[35] Q. Qian, Z. Wan, H. Takenaka, J. K. Keum, T. J. Smart, L. Wang, P. Wang, J. Zhou, H. Ren, D. Xu, *et al.*, Nat. Nanotechnol. **18**, 357 (2023).
[36] D. M. Hofmann, A. Hofstaetter, F. Leiter, H. Zhou, F. Henecker, B. K. Meyer, S. B. Orlinskii, J. Schmidt, and P. G. Baranov, Phys. Rev. Lett. **88**, 045504 (2002).
[37] K. Miyata, D. Meggiolaro, M. T. Trinh, P. P. Joshi, E. Mosconi, S. C. Jones, F. De Angelis, and X.-Y. Zhu, Sci. Adv. **3**, e1701217 (2017).
[38] K. T. Munson, E. R. Kennehan, G. S. Doucette, and J. B. Asbury, Chem **4**, 2826 (2018).
[39] M. Syperek, D. Yakovlev, I. Yugova, J. Misiewicz, I. Sedova, S. Sorokin, A. Toropov, S. Ivanov, and M. Bayer, Phys. Rev. B **84**, 085304 (2011).
[40] I. Merkulov, A. L. Efros, and M. Rosen, Phys. Rev. B **65**, 205309 (2002).
